# Negative pressure pulmonary oedema due to rigors and chills associated with liver abscess

**DOI:** 10.1002/rcr2.826

**Published:** 2021-08-21

**Authors:** Haroon Chaudhry, Swathi Nimmala, Bhavani Nagendra Papudesi, Fizza Sajjad, Sanu Paul, Zimran Gohar, Reuben Azad, Hannah Naveen, Joseph Demidovich

**Affiliations:** ^1^ Department of Internal Medicine Suburban Community Hospital East Norriton Pennsylvania USA; ^2^ Department of Science University of Albany Albany New York USA; ^3^ Department of Cardiology Albert Einstein Medical Center Philadelphia Pennsylvania USA; ^4^ Department of Medicine All Saints University School of Medicine Roseau Dominica

**Keywords:** critical care, dyspnoea, laryngeal dyskinesia, liver abscess, NPPE

## Abstract

A 61‐year‐old male presented with progressive generalized weakness, myalgia, diaphoresis, fever, episodic chills and rigors that had started 4 days previously. Chest x‐ray (CXR) showed overlying curvilinear radio‐opacities. Abdominal computed tomography revealed liver and bilateral adrenal lesions. Empiric 7‐day intravenous Piperacillin / Tazobactam (Zosyn) was initiated, and he was admitted for sepsis. After an episode of rigors on Day 2, he developed acute hypoxic respiratory failure with inspiratory stridor. CXR revealed new, bilateral airspace disease. Racemic Epinephrine, Solumedrol, Ketorolac (Toradol) and Diphenhydramine were given, and he was transferred to the intensive care unit with presumptive diagnosis of foreign body aspiration or allergic reaction. With each subsequent episode of rigor and chills, he continued developing hypoxic respiratory failure with stridor and an incremental increase in pulmonary oedema on imaging. Pulmonologist concluded it likely secondary to negative pressure pulmonary oedema caused by transient laryngeal dyskinesia induced by the increased work of breathing associated with rigors. Symptoms resolved after the complete course of antibiotics along with supportive therapy.

## Graphical abstract

Negative pressure pulmonary oedema (NPPE) is caused by upper airway obstruction and rapid negative pressure due to inspiratory efforts against an obstruction in a patient with preserved ejection fraction. NPPE is a perioperative life‐threatening emergency, but it is rarely diagnosed and reported due to unfamiliarity. Here, we discuss the case of a patient who developed acute respiratory failure with bacteraemia due to NPPE after an intense inspiratory effort against transient laryngeal dyskinesia.

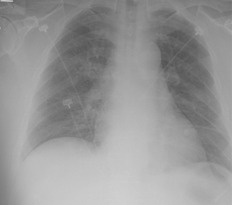

## INTRODUCTION

First reported in 1977, negative pressure pulmonary oedema (NPPE) is caused by upper airway obstruction and rapid negative pressure due to inspiratory efforts against an obstruction^1^ in a patient with preserved ejection fraction. NPPE is a perioperative life‐threatening emergency, although it has been reported as a post‐general anaesthesia complication in 0.05%–0.1% of cases.^2^ More than half of these cases are associated with post‐anaesthesia laryngospasm. As it is diagnosed rarely due to unfamiliarity, reported cases are much lower than the actual incidence. Here, we discuss the case of a patient who developed acute respiratory failure with bacteraemia due to NPPE after an intense inspiratory effort against transient laryngeal dyskinesia.

## CASE REPORT

A 61‐year‐old Asian male with a past medical history of hypertension, hyperlipidaemia and Type 2 diabetes mellitus presented with 4 days of generalized weakness, myalgias, chills, dizziness, nausea, headache, intermittent chills, rigors with profuse sweating and an episode of non‐bloody loose stool. Review of systems was negative except for above. The patient was a lifetime non‐smoker and had no prior diagnosis of any Ear, Nose, Throat (ENT) or chronic pulmonary pathology such as chronic obstructive pulmonary disease, asthma and so on.

**FIGURE 1:**
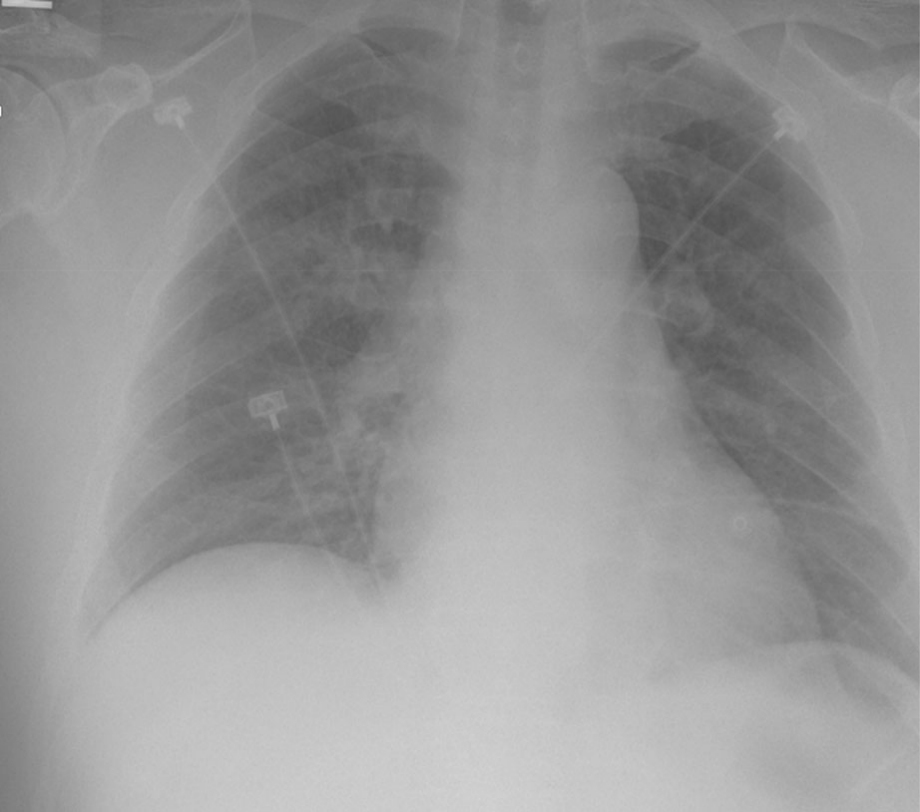
Chest x‐ray on admission Day 2 depicting incremental worsening of negative pressure pulmonary oedema with each episode of rigor and chills

On admission, vital signs were stable except for pulse oximetry (SpO_2_) 92% on room air. Comprehensive metabolic panel, complete blood count and electrocardiogram were unremarkable: lactic acid 3.3 mmol/L, arterial blood gas: pH 7.367, partial pressure of carbon dioxide (pCO_2_)35.8 mmHg, partial pressure of oxygen (pO_2_ )48.6, HCO_3_ 20.1 mmol/L, Be −4.5 mmol/L, oxygen saturation (SO_2_) 84%. Chest x‐ray (CXR) was found to have overlying curvilinear radio‐opacities (Figure 1). Abdominal computed tomography revealed lesions in the liver and bilateral adrenal glands. The patient was admitted under the working diagnosis of sepsis and started on empiric intravenous (IV) cefazolin 2 g once daily and piperacillin/tazobactam 3.375 g three times a day along with budesonide, enoxaparin, furosemide, high insulin lispro sliding scale (3–18 units at mealtimes), IV fluids and other supportive therapy.

On admission Day 1, after an episode of rigors, the patient developed dyspnoea and acute hypoxic respiratory failure with inspiratory stridor on examination. CXR revealed a new, bilateral airspace disease. Magnetic resonance imaging of the abdomen on Day 2 revealed multiple hepatic pyogenic abscesses (approximately three in the left lobes and five in the right lobes) with dominant lesion measuring 7 cm in the left hepatic lobe with a halo of oedema displaying internal restricted diffusion and thin circumferential rim and septal enhancement.

During hospitalization, the patient continued to develop hypoxic respiratory failure with stridor and incremental worsening of pulmonary oedema on CXR, with each subsequent episode of rigors and chills. Neck X‐ray demonstrated patent airway and excluded any large foreign body or retropharyngeal abscess. The NPPE exacerbation spells during hospitalization varied in intensity and were frequently associated with tachypnoea and desaturation (requiring oxygen via nasal cannula up to 6 L/min); however, the patient remained afebrile and normotensive (with white blood cells occasionally in high normal range) during these episodes. The pulmonologist assessed that NPPE was secondary to intermittent vocal cord muscle spasms associated with the patient's rigors. The patient was monitored in the intensive care unit and stepdown unit on supportive therapy.

On Day 9, a transoesophageal echocardiogram performed to rule out other serious conditions was unremarkable except for a patent foramen ovale was discovered by colour Doppler and a mildly dilated aortic root. Diagnosis of NPPE was made. It was assessed to be caused by transient laryngeal dyskinesia induced by the increased work of breathing associated with the rigors. Blood cultures later resulted positive for *Klebsiella pneumoniae*.

**FIGURE 2:**
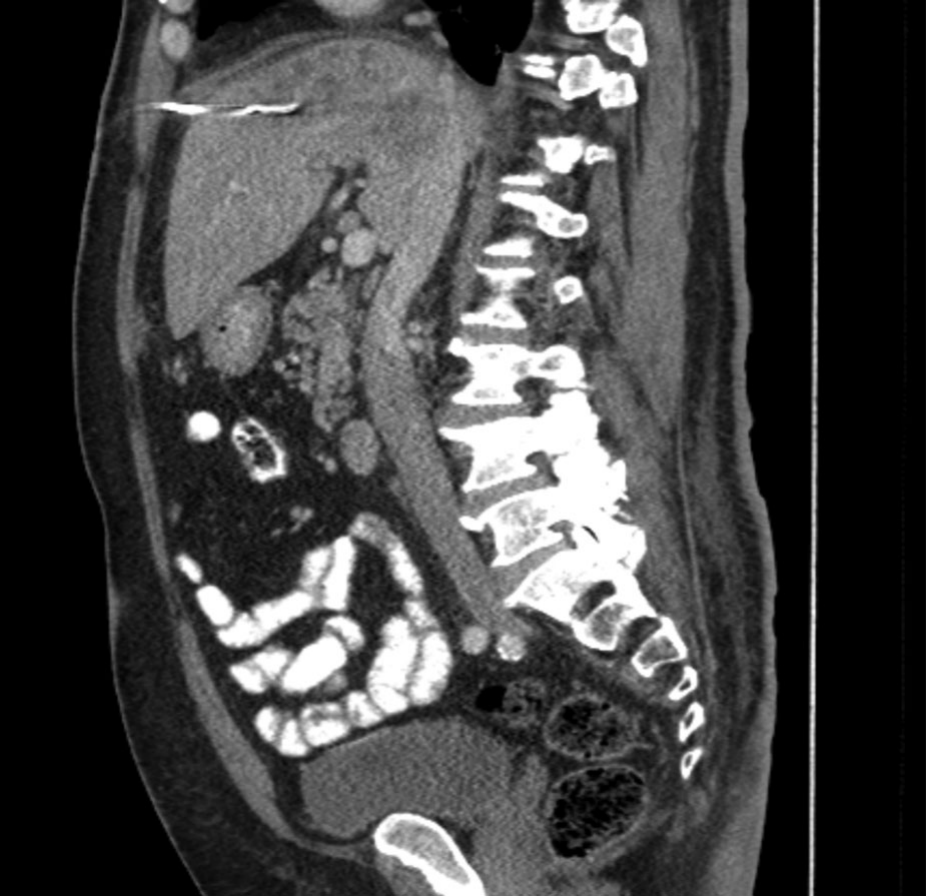
Abdominal and pelvis computed tomography with oral and intravenous contrast depicting complex loculated collection within the liver and a pigtail catheter placed (sagittal view)

On Day 10, a pigtail drainage catheter was placed to drain the largest abscess (Figure 2), which revealed mixed inflammation, degenerative cells and many *K. pneumoniae* bacteria. The respiratory failure resolved after the course of antibiotics and supportive treatment with oxygen. He was gradually weaned to room air and discharged after 10 days.

## DISCUSSION

This patient developed NPPE secondary to transient laryngeal dyskinesia during the episodes of rigors and chills in the setting of *K*. *pneumoniae*. He responded to racemic epinephrine, solumedrol, Toradol, diphenhydramine, supplemental oxygen and antibiotics. Physiologically, the net fluid transfer across the pulmonary capillaries depends on capillary permeability and the net difference between hydrostatic pressure and colloid osmotic pressure. Normally, negative pleural of −2 to −8 cm H_2_O is generated during inspiration. In NPPE, tachypnoea generates negative intrathoracic pressure as high as −140 cm H_2_O, which increases the hydrostatic pressure in the pulmonary capillary bed causing peri‐microvascular interstitial and alveolar oedema.^3^

NPPE usually develops in children and young adults immediately post‐extubation,^4^ but can be delayed up to few hours. Non‐invasive ventilation and diuretics are usually sufficient to manage NPPE patients. However, mechanical ventilation and extra‐corporal membrane oxygenation (ECMO)^5^ may be required. Physicians should be able to recognize NPPE timely for effective management of these patients.

## CONFLICT OF INTEREST

None declared.

## ETHICS STATEMENT

Appropriate written informed consent was obtained for publication of this case report and accompanying images.
